# Clinical outcomes and survival comparison between NexGen all-poly and its metal-backed equivalent in total knee arthroplasty

**DOI:** 10.1007/s00264-023-05772-3

**Published:** 2023-04-18

**Authors:** Vasileios Apostolopoulos, Luboš Nachtnebl, Michal Mahdal, Lukáš Pazourek, Petr Boháč, Pavel Janíček, Tomáš Tomáš

**Affiliations:** 1 First Department of Orthopaedic Surgery, St. Anne’s University Hospital and Faculty of Medicine, Masaryk University, FN u Sv Anny Brne, Pekarska 53, Brno, 60200 Czechia; 2 Institute of Solid Mechanics, Mechatronics and Biomechanics, Faculty of Mechanical Engineering, University of Technology, Brno, Czechia

**Keywords:** Knee arthroplasty, All-polyethylene knee replacement, NexGen, Implant survival, Knee Society Score

## Abstract

**Purpose:**

This study aims to compare total knee replacement (TKA) with NexGen All-Poly (APT) and NexGen Metal-Backed (MBT) in terms of implant survivorship, reasons leading to implant failure and functional results of defined age categories.

**Methods:**

A single-centre, retrospective evaluation of 812 patients who underwent knee replacement with NexGen CR between 2005 and 2021, comparing a modern congruent APT component to a modular MBT equivalent component using a similar surgical technique at a notable mean follow-up duration. Implant survival, functional outcomes using the Knee Society Score and range of motion were evaluated and compared in different age categories.

**Results:**

Of the 812 NexGen CR TKAs performed at our institution, 410 (50.4%) used APT components and 402 (49.6%) MBT components. The survival rate of NexGen APT was 97.1% and that of NexGen MBT was 93.2% (*p* = 0.36). Removal of the implant occurred overall in 15 cases, for MBT in ten cases, and for APT in four cases. The FS was proved to be significantly higher when APT components were implanted in younger patients than for MBT (*p* = 0.005). A similar range of motion between the components was recorded (*p* = 0.1926).

**Conclusion:**

Under defined conditions, we measured the clinical results of implants from a single manufacturer implanted in a single department using a similar surgical technique. Considering the limitations, we suggest that all-polyethylene tibial components are equal or even superior to metal-backed ones across the examined age categories.

## Introduction

Total knee arthroplasty (TKA) is considered to be a highly effective procedure and a definitive solution for severe degenerative knee arthritis. In recent decades, most total knee replacements have been performed with modular metal-backed tibial (MBT) components [1]. All-polyethylene tibial (APT) implants are primarily recommended for older and low-demand patients [2]. Nevertheless, clinical evidence has shown no significant differences between APT and MBT. The available literature indicates that the two implants have similar results in assessing survivorship and functional outcomes [3]. However, the use of APT in primary TKA is regaining interest considering the economic strain on health care.

One of the main factors affecting clinical outcomes is the age at implantation [4]. As MBT and APT TKAs are primarily recommended for different age categories, this factor needs to be carefully monitored. Also, in the available literature, clinical comparison in younger patients has not been specifically done. Previous biomechanical analysis on APT demonstrated, using the finite element method, that APT in patients of the 60- and 70-year age groups showed a similar induced mechanical response. Moreover, APT was shown to induce remodelling and modelling of the periprosthetic tibia which is a beneficial factor in implant survivorship. As a result, more frequent implantation of APT in younger patients was suggested [5].

The NexGen TKA has been used worldwide for more than 20 years. Components of the NexGen Knee System have achieved some of the lowest revision rates [6, 7]. The NexGen CR prostheses have a similar geometric design in both the MBT and the APT versions. Despite numerous studies that have compared APT with MBT, only a few analysed TKA from a single manufacturer implanted in a single-orthopaedic department using the same surgical technique [8]. This study was conducted to retrospectively compare the clinical outcomes between NexGen CR all-poly- and TKA NexGen CR metal-backed TKAs. The purpose of this comparison is to focus on implant survivorship, the clinical results of different age categories and the reasons leading to implant failure.

## Methods

### Population

**Fig. 1:**
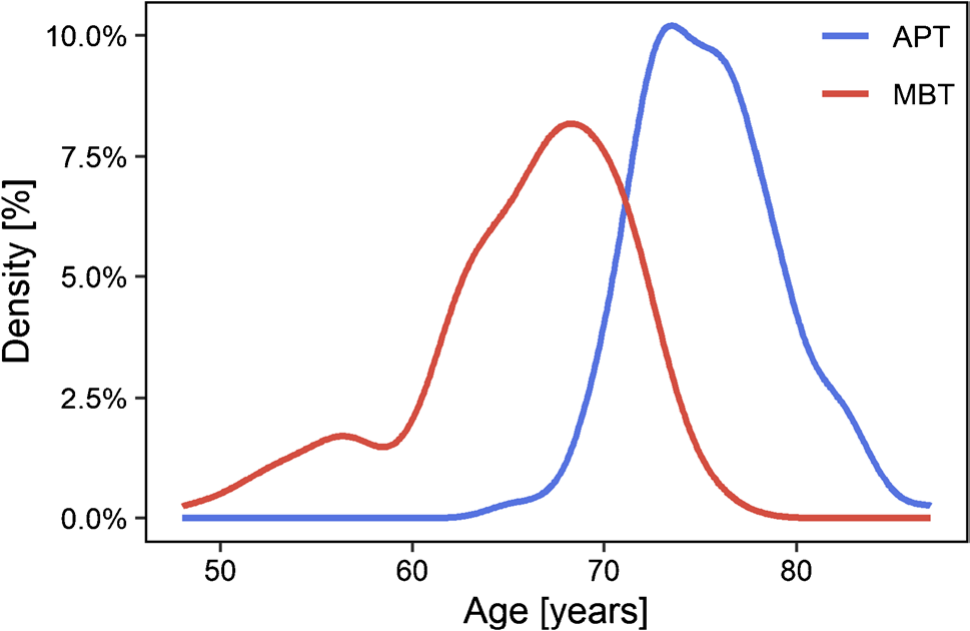
Patient age on the day of implantation of NexGen APT and MBT TKA in our institution from 2005 to 2021

For this retrospective study, survival data of 812 NexGen CR TKA implants were selected. During the period from January 2005 to May 2021, 410 total knee replacements using NexGen CR all-poly implants and 402 total knee replacements using NexGen CR metal-backed implants were performed at our institution. There were 432 females and 376 males. Patients with implanted NexGen TKA bilaterally were excluded from the study. The mean age at implantation (Fig. 1) was 70.1 years (median 71 years) overall, 75.4 years for patients treated with APT and 65.9 years for patients treated with MBT (*p* = 0.001).

Patient consent was collected pre-operatively after they were informed of the procedure and was following the principles of the Declaration of Helsinki.

### Statistical analysis

Kaplan–Meier survival analysis was used to compare implant survivorship between NexGen CR all-poly and NexGen CR metal backed. Survival analysis was done in a monitored 12-year period.

Three hundred sixteen patients were followed for at least three years, from three to 17 years, and the mean follow-up was 5.88 (± 3.1) years (Table 1). In the evaluated group, 175 patients with APT and 141 patients with MBT were recorded and their functional and radiological outcomes were analysed.

**Table 1:** Implant type, age category characteristic and follow-up of patients with NexGen TKA in our institution

Variable	APT	MBT
*N*	141 (44.6%)	175 (55.4%)
Mean follow-up	6.01 years	5.72 years
Median follow-up	5 years	5 years
≤ 71 years	17 (5.38%)	154 (48.73%)
> 71 years	124 (39.24%)	21 (6.65%)

To emphasise the results in terms of age at implantation, based on the median age (71 years), each implant dataset was divided into patients older than 71 years (referred to in the results as O) and patients 71 years old or younger (referred to in the results as Υ). One hundred twenty-four patients treated with APT were older than 71 years and 17 patients were 71 or younger. Analogically, 21 patients treated with MBT were older than 71 years and 154 were 71 or younger (Table 1).

### Evaluation

The primary efficacy endpoint was to compare the clinical and social quality of life as per the Knee Society Score which was assessed pre-operatively at the three year follow-up and eventually modified at the last follow-up. The secondary efficacy endpoint was to determine the maximum range of motion in patients observed at a minimum of three year follow-up after the surgery.

### Surgical technique

The same surgical technique as the standard TKA CR implantation method practised at our institution was applied in all cases. A midline longitudinal skin incision and medial parapatellar approach to the knee were used. The mechanical alignment technique method was chosen using the NexGen instrumentation. Bone cement was spread on the cut surface of the tibia and femur, and also on the implant itself. Patellar resurfacing was done in some cases; patellar denervation with electrocautery and osteophyte removal were done in all cases.

### Software and statistical tests

Statistical analysis was done using R software (version 4.0.5) in the RStudio development environment. Kaplan–Meier analysis was used for the overall evaluation of survival, and the Cox proportional hazard model was used to include the adjustment variable age. Fisher’s exact test was used to evaluate the dependence of two categorical variables. The Mann–Whitney test was used to evaluate the difference between two groups for a continuous variable. Fisher’s exact probability test was used to compare the proportions between the two groups.

## Results

### Implant survival

**Fig. 2:**
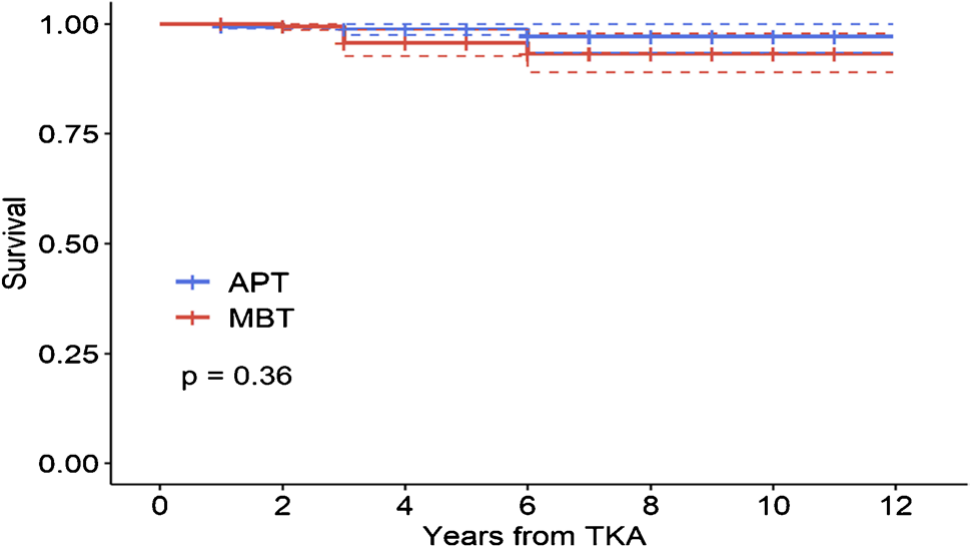
Cumulative 12-year survival curve of NexGen CR all-poly- and CR metal-backed TKAs in our institution from 2005 to 2021

The overall 12-year survival rate calculated, from a total dataset of 812 NexGen TKAs, using the Kaplan–Meier method was 94.9%. The survival rate of NexGen CR All-Poly was 97.1% and that of NexGen CR metal-backed was 93.2% (Fig. 2). Removal of the implant occurred overall in 15 cases, for MBT in 10 cases, and for APT in 4 cases (Table 2). Not considering the adjustment variable age at implantation, there was no statistically significant relationship between the survival rate and the type of implant (*p* = 0.36).

**Table 2:** Causes of NexGen TKA removal in our institution from 2005 to 2021

Cause of removal	Total	APT, *N* = 410	MBT, *N* = 402	*P* value
Infection	4	2 (0.49%)	2 (0.5%)	> 0.99
Periprosthetic fracture	2	1 (0.24%)	1 (0.25%)	> 0.99
Aseptic loosening	2	0 (0%)	2 (0.5%)	0.246
Instability	3	0 (0%)	3* (0.75%)	0.1223
Tumour	1	0 (0%)	1 (0.25%)	0.4957
Femoral component overstuffing	1	1 (0.24%)	0 (0%)	> 0.99
Plateau exchange	2	0 (0%)	2* (0.5%)	0.246

*One case of plateau exchange due to instability

Reimplantation of the tibial component for aseptic loosening or instability was not necessary in any APT case, compared to two cases of MBT (*p* = 0.246). There was only one case of TKA instability with APT because of tibia component valgisation but it was not revised due to the poor health status of the patient. Also, there was one case recorded of TKA removal due to renal cell tumour metastasis that caused osteolysis of the proximal tibia. Twenty-one patients were followed up for more than 12 years (max. 17 years). Those results are excluded from the showing examination because of the small sample size.

A dataset of 316 patients completed a minimum of 3-year follow-up and their results were evaluated. The overall mean follow-up was 5.88 (± 3.1) years. Moreover, a similar follow-up between the implant categories was recorded (*p* = 0.169).

To include the adjustment variable age at implantation in the survival analysis, instead of Kaplan–Meier analysis, we used the Cox proportional hazard model in the monitored 12-year period. Considering the median age of 71 years, *p* = 0.026 and Exp(coef) shows the failure risk is 18.4. We can interpret the value of 18.4 by MBT patients in our cohort compared to patients with APT having a significantly higher risk of the occurrence of a failure, given that we have a fixed patient age.

### Functional outcomes

**Fig. 3:**
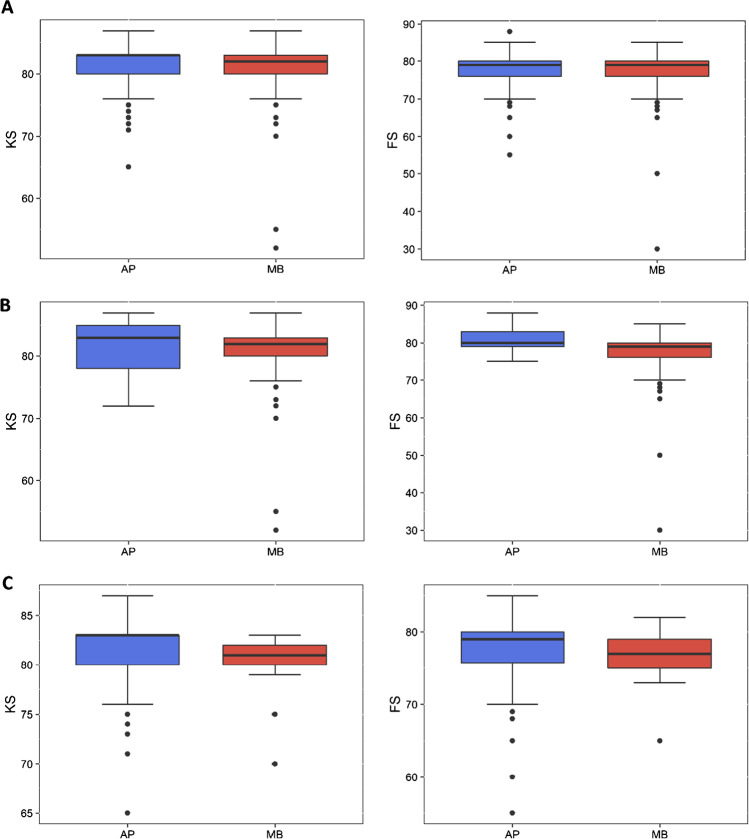
Knee Society Score of patients with NexGen CR all-poly- and CR metal-backed TKAs in our institution after the 3-year follow-up. **A** Overall. **B** Patients 71 years old or younger. **C** Patients older than 71 years

The mean measured KS (knee score) of patients with APT was 81.51 (± 3.4) and that of patients with MBT was 81.19 (± 4.23; *p* = 0.399). The mean measured FS (function score) of patients with APT was 77.65 (± 4.57) and that of patients with MBT was 77.41 (± 5.53; *p* = 0.717) (Fig. 3).

The mean measured KS of patients 71 years old or younger with APT was 81.94 (± 4.28) and that of younger patients with MBT was 81.31 (± 4.36; *p* = 0.310). However, patients 71 years old or younger with APT had a mean FS of 80.71 (± 3.55) and those with MBT had a mean FS of 77.5 (± 5.75); the difference was statistically significant (*p* = 0.005) (Fig. 3).

The mean measured KS of patients older than 71 years with APT was 81.45 (± 3.28) and that of older patients with MBT was 80.29 (± 2.99); the difference has been proved to be statistically significant (*p* = 0.029). Also, patients older than 71 years with APT had a mean FS of 77.23 (± 4.55) and those with MBT had a mean FS of 76.76 (± 3.53); the difference was not statistically significant (*p* = 0.215) (Fig. 3).

### Range of motion

**Fig. 4:**
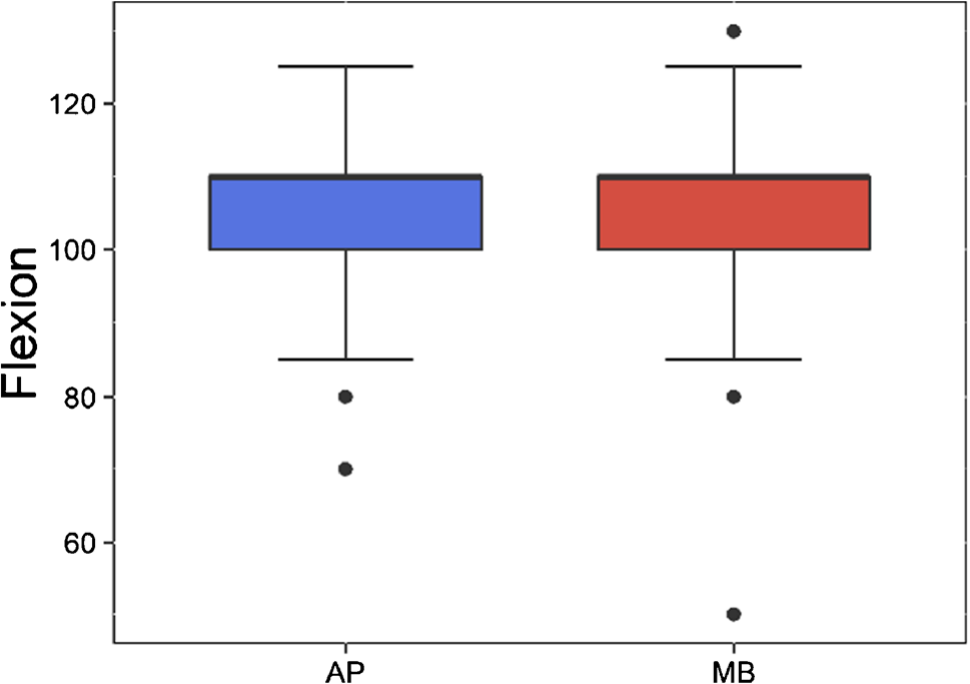
Range of motion of patients with NexGen CR all-poly- and CR metal-backed TKAs in our institution at the 3-year follow-up

The mean knee flexion angle of patients with APT was 105.5 (± 10.93) degrees, and the mean flexion contracture was 6.43 (± 2.44) degrees and occurred in seven cases. On the contrary, the mean knee flexion angle of patients with MBT was 107.3 (± 10.07) degrees (*p* = 0.1926), and the mean flexion contracture was 6.43 (± 2.44) degrees and occurred only in seven cases (Fig. 4). Manipulation under anaesthesia was necessary in 11 cases because of stiffness, five cases with MBT and 6 cases with APT. No cases were treated with arthroscopic or open lysis of adhesions.

## Discussion

Rates of total joint replacement are increasing significantly [9]. In modern orthopaedics, MBT TKA implantation is preferred to APT. According to Scandinavian arthroplasty registries, only 0.1–13% of TKAs use APT [8, 10]. Considering that APT is notably cheaper than MBT equivalents, regardless of the manufacturer, a more frequent APT implantation could be cost-saving for the healthcare system [11]. A recent study showed that the use of an APT implant can significantly affect the surgical cost and also the total hospital admission cost [12]. Furthermore, the majority of studies comparing APT and MBT implants have shown no difference in clinical results. Our study demonstrates equal clinical outcomes and survivorship even in younger patients. As a result, the use of APT could be cost-effective in avoiding worse outcomes or higher revision rates even in younger patients. This fact may support the use of the cheaper but reliable implant, especially where economical burdens affect implant selection considering the difference in cost.

Although there have been numerous clinical studies, there are only a few clinical studies examining survivorship and functional outcome of TKA equivalents of a similar design from one manufacturer, implanted in a single-orthopaedic department. A registry study compares APT and MBT from a single manufacturer, but results from numerous institutes with different standards were analysed [13]. Our cohort includes only patients implanted with NexGen TKAs in our department. As a result, the surgical technique used was similar in all cases, consistent with the standard implantation method, and the manufacturer’s instructions were followed. To achieve full-fledged results, the measured Knee Society Score was observed at a minimum of 3-year follow-up, excluding patients with shorter follow-up from functional comparison.

With 5.88 years of mean follow-up, the NexGen implants with all-polyethylene and metal-backed tibial components were identified as equal in terms of clinical outcome. The 12-year survival rate of the APT implant was 97.1%. During the same period, knee replacements with the metal-backed equivalent had a slightly lower survival rate of 93.2%. For seven year survival, the APT component achieved a high survival rate of 97.1%. Similar medium-term results of APT were described by Selvan et al. [14]. Another study comparing early- to mid-term clinical survivorship of 1064 implants described superior results for patients with APT [15]. In our study, APT components were found to have a lower rate of all-cause revision, tibial component loosening and periprosthetic fracture. Reimplantation of the tibial component due to aseptic loosening was not necessary in any of the 410 APT cases.

One of the most highlighted advantages of MBT is considered to be the modularity and the possibility of polyethylene insert exchange [16]. From our cohort of 406 patients with MBT, polyethylene insert exchange was necessary only in two cases. In the first case, the insert was exchanged for one 4 mm higher due to anteroposterior instability. In the second case, there was a polyethylene insert dislocation. There was no case of polyethylene insert exchange due to wear by abrasion of the previous insert.

In our paper, we report similar clinical results between the two implants in terms of the range of motion. A slightly greater range of motion was measured in patients with MBT, which could be explained by the younger mean age of those patients at implantation compared to APT. The difference in range of motion was not found to be significant. A similar range of motion between APT and MBT was also recorded in a long-term implant comparison [17].

To measure clinical outcome, we used one of the most commonly reported instruments, the Knee Society Score [18]. Numerous up-to-date studies have recorded their post-operative results using the Knee Society Score [19, 20]. The mean KS of the implants was similar (APT 81.51 and MBT 81.19), showing no statistically significant difference between the 2 implants. Using the same scoring system, a systematic review registered analogical implant functional performance [3].

A previous study demonstrated a lower all-cause risk of revision, in patients younger than 65 years old, when APT was implanted, compared to MBT [13]. Houdek et al. reviewed 31,939 patients from a 43-year period, recording superior survivorship of APT, regardless of the age group, even when APT was implanted in younger patients [21]. In our age-specific analysis, the FS was proved to be significantly higher when APT components were implanted in younger patients than for MBT (77.5). This finding will be the subject of further investigation in future works. On the other hand, no significant differences were registered in the mean measured KS between the age groups. This could be explained because the KS is more dependent on the surgical technique and less associated with patient infirmity. The only exception was recorded in the KS of patients older than 71 years; then, the APT component was proved to be superior.

The strengths of this study include its large sample size, the use of implants from a single manufacturer (Zimmer) comparing a modern congruent APT component to a modular MBT component of the same design, the similar surgical technique and the notable mean follow-up duration. Those attributes allowed an assessment and comparison of the implants excluding variables that could negatively affect the validity of the results. On the contrary, the main limitation is the design of the study. This is a retrospective study with a cohort of patients with wide variance in follow-up. Also, there were significantly different ages at implantation. To achieve the adjustment variable age, the Cox proportional hazard model was used with a fixed age at implantation. Even though a similar surgical technique was performed, different surgeons led the procedure. Considering the limitations, we suggest that relevant results were obtained.

## Conclusion

In summary, we have compared the survival and the functional outcome of the NexGen all-polyethylene tibial component with the equivalent metal-backed component. Under defined conditions, we measured the clinical results of implants from a single manufacturer implanted in a single department using a similar surgical technique. Considering the limitations, we suggest that APT components are equal or even superior to metal-backed ones across the age categories. The results of our study could lead to more frequent implantation of TKAs with APT components, as a cost-effective option, even in younger patients.

## Data Availability

The data that support the findings of this study are available from the corresponding author upon reasonable request.
